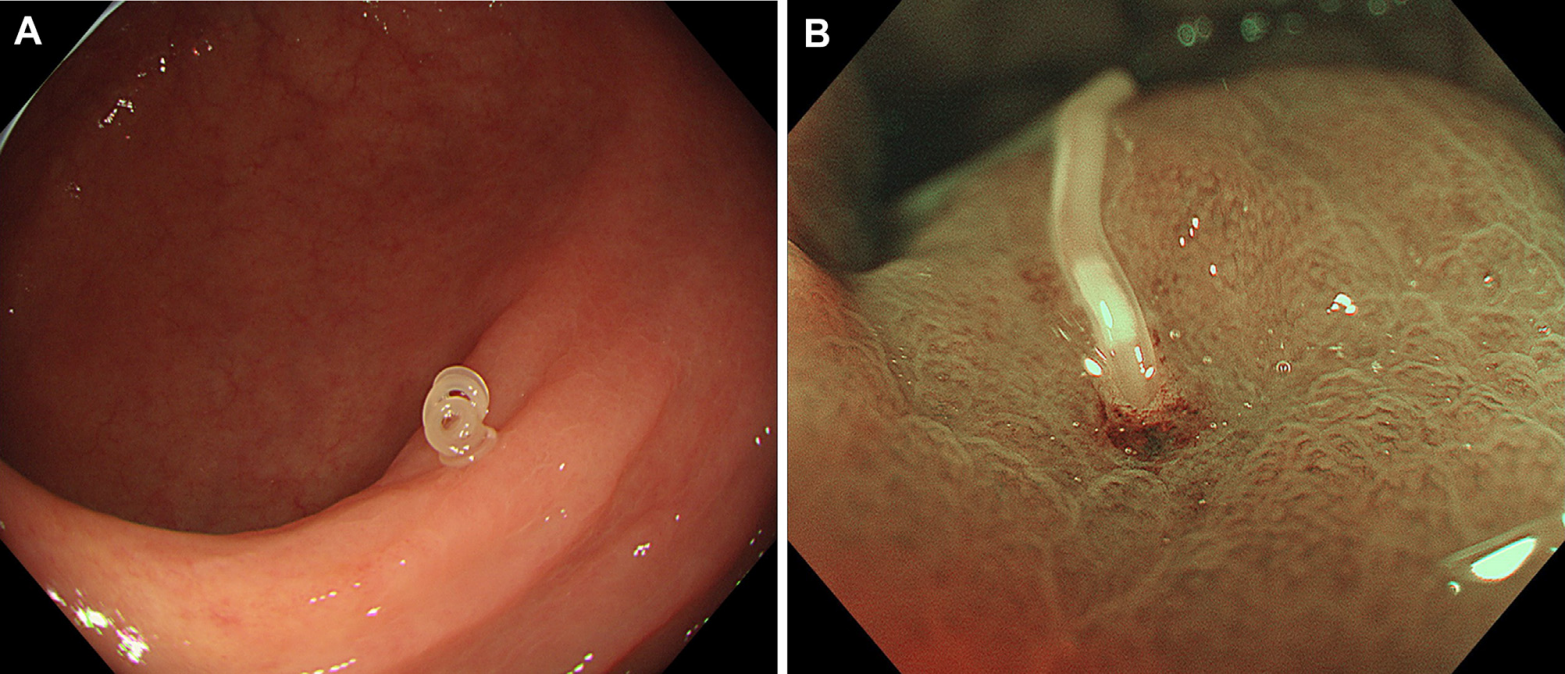# Colonic Anisakiasis

**DOI:** 10.1016/j.gastha.2022.08.010

**Published:** 2022-09-07

**Authors:** Tomohiko Mannami, Genyo Ikeda, Nobukiyo Fujiwara

**Affiliations:** 1 Department of Gastroenterology, National Hospital Organization Okayama Medical Center, Okayama, Japan; 2 Department of Internal Medicine, Chugoku Central Hospital, Fukuyama, Japan

A 75-year-old man with hypertension, who was otherwise healthy, underwent a surveillance colonoscopy 1 year after a polypectomy. He had no specific symptoms, and his vital signs were normal. The examination showed an *Anisakis* larva invading the mucosa of the proximal ascending colon, where a semilunar fold was edematous and thickened with a small erosion on the inserted site (Figure A). Magnifying endoscopy with narrow-band imaging revealed a small whitish elongated spot (the ventricle, an organ located between the esophagus and the intestine of Anisakis larva), which was more clearly visible than with conventional white-light endoscopy (Figure B). The larva was removed using biopsy forceps, and no symptoms were seen after the colonoscopy. The patient revealed that he had eaten sushi, sashimi, and shime saba (vinegared mackerel) 4–5 days before the colonoscopy.

Anisakiasis occurs most frequently in the stomach, with less than 1% of gastrointestinal anisakiasis cases identified in the large intestine. Chronic anisakiasis of the colon can lead to development of abscesses and granulomas, which may cause intussusception or be misdiagnosed as cancer. Therefore, endoscopic removal is recommended even when encountered incidentally in asymptomatic cases.

**Figure:**